# Multimodal biological profiles of symptom-based subgroups in recent-onset psychosis

**DOI:** 10.21203/rs.3.rs-7867983/v1

**Published:** 2025-10-30

**Authors:** Nicholaos Koutsouleris, Madalina O. Buciuman, Clara Vetter, Ananda Bajrić, Clara Weyer, Santiago Tovar Perdomo, Adyasha Tejaswi Khuntia, yuri milaneschi, David Popovic, Dominic Dwyer, Katharine Chisholm, Lana Kambeitz-Ilankovic, Linda Antonucci, Theresa Lichtenstein, Marlene Rosen, Stephan Ruhrmann, Joseph Kambeitz, Anita Riecher-Rössler, Rachel Upthegrove, Raimo Salokangas, Jarmo Hietala, Christos Pantelis, Rebekka Lencer, Eva Meisenzahl, Stephen Wood, Paolo Brambilla, Stefan Borgwardt, Alessandro Bertolino, Peter Falkai

**Affiliations:** Ludwig Maximillian University; Department of Psychiatry and Psychotherapy, Ludwig-Maximilian-University, Munich; Department of Psychiatry and Psychotherapy, Ludwig-Maximilian-University, Munich; Max Planck School of Cognition, Leipzig; Department of Psychiatry and Psychotherapy, Ludwig-Maximilian-University, Munich; Department of Psychiatry and Psychotherapy, Ludwig-Maximilian-University, Munich; Ludwig-Maximilian-University; Amsterdam UMC, Vrije Universiteit/GGZ inGeest; Max Planck Institute of Psychiatry; Centre for Youth Mental Health, University of Melbourne; School of Psychology, University of Sussex; Department of Psychiatry and Psychotherapy, Faculty of Medicine and University Hospital, University of Cologne, Cologne; Department of Translational Biomedicine and Neuroscience, University of Bari Aldo Moro; Department of Psychiatry and Psychotherapy, Faculty of Medicine and University Hospital of Cologne; Department of Psychiatry and Psychotherapy, Faculty of Medicine and University Hospital of Cologne; University of Cologne, Faculty of Medicine and University Hospital; Faculty of Medicine and University Hospital University of Cologne, Cologne; Medical Faculty, University of Basel; University of Oxford; Department of Psychiatry, University of Turku; University of Turku; University of Melbourne; Department of Psychiatry and Psychotherapy, University of Münster; Department of Psychiatry and Psychotherapy, Medical Faculty, Heinrich-Heine University; University of Melbourne; University of Milan; University of Lübeck; Department of Translational Biomedicine and Neuroscience, University of Bari “Aldo Moro”, Bari, Italy; Ludwig Maximilians University Munich

**Keywords:** neuroimaging, polygenic risk score, neurocognition, factor analysis, early psychosis, subtyping

## Abstract

Symptom diversity in psychoses complicates the search for biological markers. Using Positive and Negative Symptom Scale data from 362 recent-onset patients in the multicenter PRONIA study, we identified four subgroups with dominant symptom patterns: motor/cognitive, positive, social withdrawal, and affective. Subgroups were compared against each other and to 338 healthy controls across neurocognition, brain imaging, and genetic risk. Patient subgroups shared a profile of impaired processing speed and altered functional connectivity, with increased coupling in sensorimotor networks and reduced connectivity within and between default-mode, salience, and control networks. Variations in modality-specific neurobiological underpinnings differentiated subgroups, with fronto-temporal gray-matter loss characterizing the motor/cognitive and positive subgroups, and elevated genetic risk best separating the positive subgroup from the rest. The motor/cognitive group showed the most severe alterations across modalities, reaching the highest multimodal separability from controls (Balanced Accuracy=82.1%, sensitivity=74.9%, specificity=89.3%). Our findings support a framework of shared biological dysfunction with modality-selective vulnerabilities shaping symptom heterogeneity in early-stage psychotic disorders.

## Introduction

Psychosis is an umbrella term for diverse and dynamically evolving clinical phenotypes, complicating the development of more precise, biologically informed diagnostic and therapeutic tools[1], [2]. Traditional psychiatric classifications, such as the Diagnostic and Statistical Manual of Mental Disorders (DSM [3]), are increasingly complemented by frameworks like the Research Domain Criteria (RDoC [4]) or the Hierarchical Taxonomy Of Psychopathology (HiTOP [5]), highlighting symptom-based dimensions potentially more closely aligned to underlying neurobiological mechanisms.

In schizophrenia, the distinction between positive (e.g., hallucinations, delusions), negative (e.g., blunted affect, avolition, anhedonia), and cognitive symptoms (e.g., disorganized speech and thought) has proven helpful in delineating the symptom complexity observed in clinical practice [6], [7], based on scales such as the *Positive and Negative Syndrome Scale* (PANSS, [8]). In early psychosis, four to five PANSS symptoms-based subgroups have been reported [7], [9]–[14], with symptom dimensions shown to dynamically evolve [15], [16]) and associate with functional ([10], [14]) and treatment outcomes [11].

Distinct symptom dimensions have been associated with different layers of biological variability, suggesting differential alignment with pathophysiological processes. Neurocognitive functions closely linked to specific brain networks [17]–[19] are mainly associated with negative symptoms in early-stage psychosis [20]–[23]. Relatedly, resting-state functional MRI (rs-fMRI) abnormalities have been associated with negative [24], positive [25], [26] and disorganized [27], [28] symptoms in schizophrenia and first-episode psychosis, with distinct and even opposite associations being reported for these symptom dimensions [29], [30]. Furthermore, symptom-dependent structural brain abnormalities have been reported in patients with schizophrenia [31]–[34] or first-episode psychoses [35]–[41], with both distinct and common prefrontal-perisylvian gray matter volume (GMV) alterations characterizing patients with predominantly positive, negative, or disorganized symptoms [31]. Similarly, the genetic liability for schizophrenia has been associated with symptom dimensions both in chronic [42], [43] and first-episode stages of psychotic disorders [44], [45]. Specifically, significant associations between polygenic risk scores for schizophrenia and negative [45], positive [45], and general PANSS-based symptoms [44] were reported in first-episode psychoses, highlighting the complex associations between genetic factors and symptom constellations.

Collectively, existing evidence points to both shared and distinct biological signatures across psychotic symptom dimensions, underscoring the need for a comprehensive, multilayer characterization of symptom-defined subgroups. In this context, machine learning tools are well-suited to integrate high-dimensional, cross-modal signals and reveal complex neurobiological pathways that pleiotropically map to psychosis. Here, we applied this framework in the multi-site European PRONIA cohort (recent-onset psychosis, ROP; https://www.proniapredictors.eu/pronia/index.html) by: (1) deriving the PANSS-based symptom structure within ROP patients and its longitudinal stability over 9- and 18-month follow-up time points using a data-driven factorization approach, (2) training machine learning models to differentiate patient subgroups defined by their most pronounced symptom dimension from each other and against healthy control (HC) individuals based on neurocognition, rs-fMRI measures, GMV, and polygenic risk scores (PRS) to obtain a deep multimodal delineation of disease heterogeneity in early illness stages, and (3) comparing longitudinal functioning trajectories of the obtained patient subgroups to assess their prognostic value.

## Results

### Optimal factor solution

At T0, a four-factor model outperformed other solutions as determined by jackknife resampling (*BIC*[95%-CI]=−1207[–1208,–1207]; see Figure S2). The model’s factors yielded cumulative variances of *s*=13%, 22%, 30%, and 37%. The items with the highest loading on each factor were “lack of spontaneity”, “blunted affect”, “poor rapport”, and “motor retardation” on the first factor (in the following referred to as *Motor/Cognition* factor); “lack of judgment and insight”, ”unusual thought content”, “excitement”, and “delusions” on the second factor (*Positive* factor); “passive/apathetic social withdrawal”, “active social avoidance”, “emotional withdrawal”, and “disturbance of volition” on the third factor (*Social Withdrawal* factor); and “anxiety”, “tension”, “depression”, and “guilt feelings” on the fourth factor (*Affective* factor; see Table S6). The internal consistencies of the four symptom factors were a_standardized_=.84, .75, .74, and .66. For factor correlations and factor score correlations, see Table S7 and S8. We grouped patients based on their maximum factor loadings (see Figure S3) into four subgroups: ROP-MOTCOG (*Motor/Cognition*, N=92), ROP-POS (*Positive*, N=92), ROP-SOCWD (*Social Withdrawal*, N=82), and ROP-AFF (*Affective*, N=96). Mean assignment certainty quantified using jackknife resampling by subgroup was: ROP-MOTCOG: 95.5 (SD=7.4%); ROP-POS: 92.5 (SD=11.4%); ROP-SOCWD: 95.7 (SD=7.7%); ROP-AFF: 93.1 (SD=11.8%). Overall mean certainty was 94.1 (SD=9.97%). Among patients initially categorized as ROP-MOTCOG with available T1 data (N=54), 59.26% remained ROP-MOTCOG at follow-up; among those with available T2 data (N=23), 60.87% remained stable. Among patients initially categorized as ROP-POS with available T1 data (N=61), 32.79% remained ROP-POS; among those with available T2 data (N=39), 23.08% remained stable. Among patients initially categorized as ROP-SOCWD with available T1 data (N=43), 27.91% remained in the ROP-SOCWD group; among those with available T2 data (N=30), 30.00% remained stable. Of the patients initially categorized as ROP-AFF with available T1 data (N=64), 40.62% remained ROP-AFF; among those with available T2 data (N=44), 34.09% remained stable. For a full overview of between-subgroup shifts, see Table S9. Table S10 shows the correlations of factor scores between time points.

### Sociodemographic and Clinical Group-Level Comparisons

No statistically significant differences emerged between ROP subgroups and HC as well as between ROP subgroups for any sociodemographic, clinical characteristic (all FDR-corrected *P*>.05; Table 1), or distribution of ICD-10 diagnoses (χ^2^(24)=32.5, *P*=.12; Figure S4).

### Multivariate biological signatures of ROP subgroups

#### Neurocognitive models

**Figure 1:**
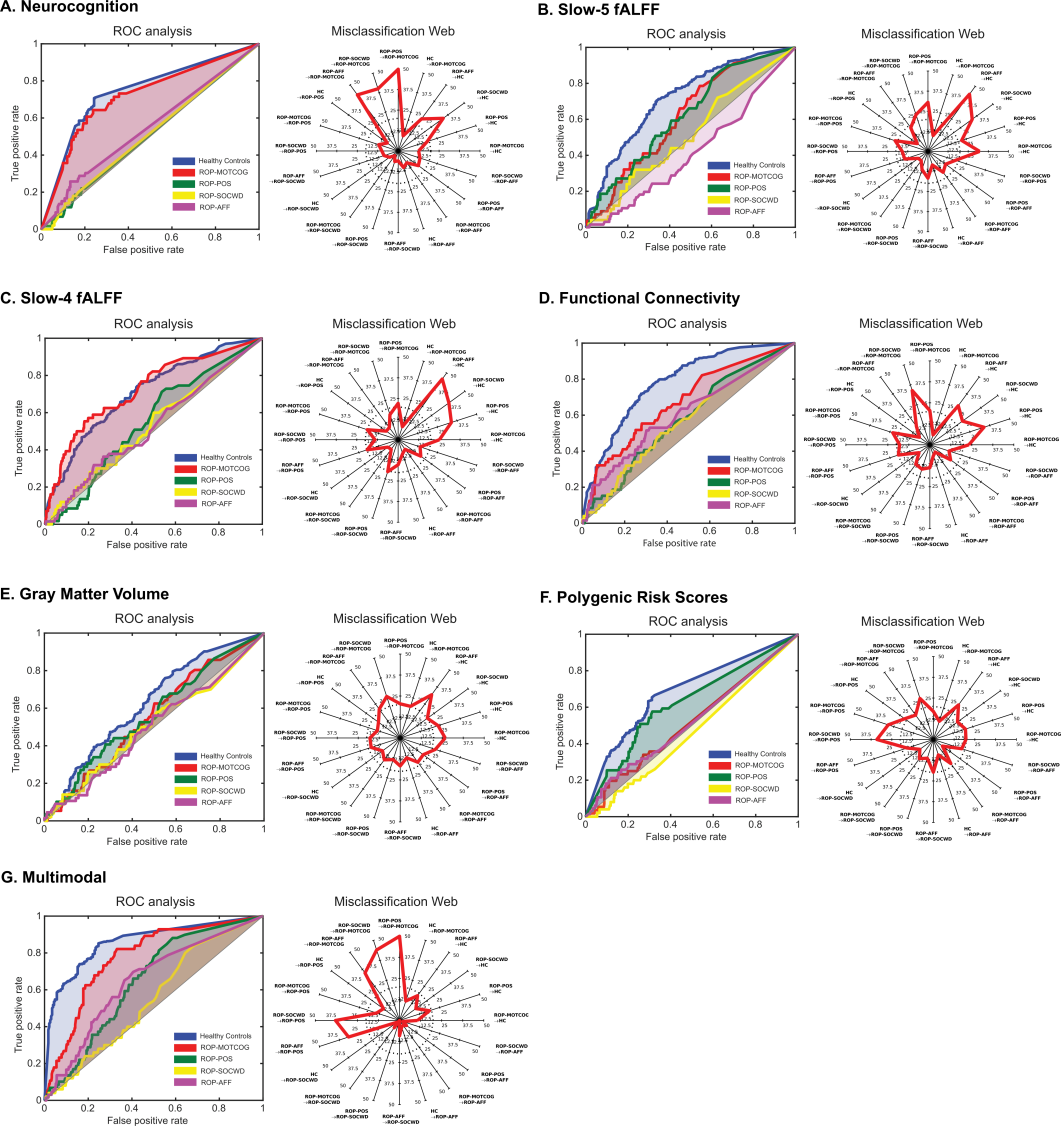
Visualization of multi-class uni- and multimodal machine learning results. For each data modality (**A-G**), the **left panel** represents the receiver operating curves (ROC) for the averaged one-vs-rest group comparisons (average of binary one-vs-one classifications) for healthy controls and each of the symptom-based ROP subgroups. The **right panels** represent the misclassification rates for each of the group comparisons within the multi-class context.

In the multi-class one-vs-rest modelling, neurocognitive variables best separated the ROP-MOTCOG subgroup from the rest of the patients and HC, while the other ROP subgroups showed poor discriminability (Figure 1A and Table S11).

**Figure 2:**
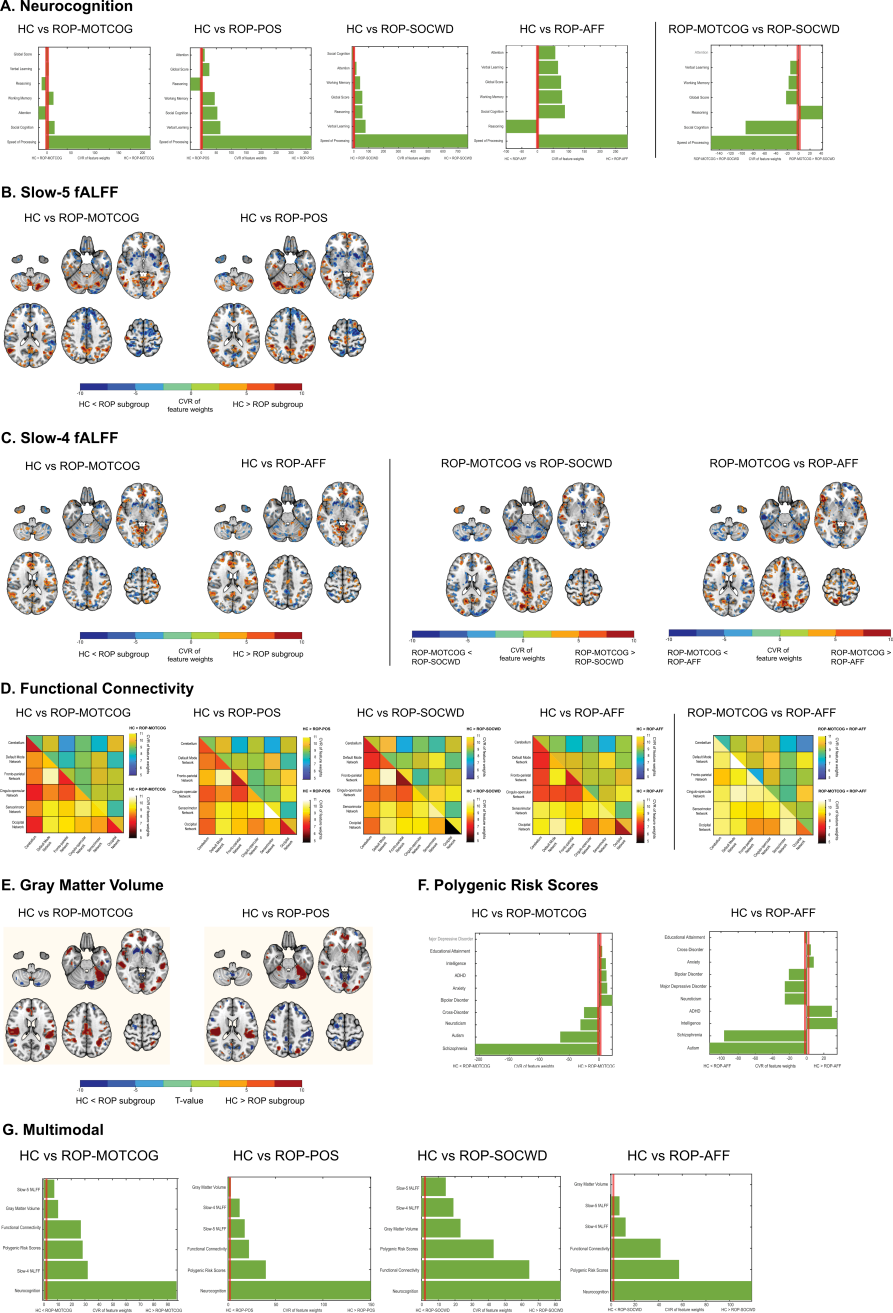
Predictive patterns of one-vs-one models within the multi-class uni- and multimodal machine learning classification framework. For each modality (A–G), columns show pairwise contrasts among HC and ROP subgroups (MOTCOG, POS, SOCWD, AFF). We display only classifiers that exceeded chance level in permutation tests (see Table 2). Feature stability across folds was indexed by the cross-validation ratio (CVR). Shown features/voxels met FDR-corrected significance at α=.05 using a sign-based consistency test for feature importance [82]. Specifically, sign-based consistency is computed as the times that a specific feature maintains the same sign (positive/negative) across the ensemble, producing a consistency between 0 (equally positive and negative weights across the ensemble) and 1 (perfect sign consistency), based on which z-scores and associated normal cumulative distribution-based standard *P* values for each feature are derived.

Considering one-vs-one models, neurocognition differentiated HC from all ROP sugroups (BAC range: [62.7% - 77.5%], see Table 2), with speed of processing showing the strongest deviation from HC across groups (Figure 2A).

Additionally, neurocognitive models differentiated the ROP-MOTCOG subgroup from ROP-SOCWD (BAC=60.2%, sensitivity=80.4%, specificity=40.0%), with the ROP-MOTCOG group showing reduced cognitive abilities across most domains in comparison to ROP-SOCWD (Figure 2A).

#### Resting-state fMRI models

The multi-class results revealed the highest separability for the ROP-MOTCOG versus the other participants across rs-fMRI modalities (Figure 1B/C/D, Table S11).

At one-vs-one level, slow-5 fALFF models significantly differentiated HC from ROP-MOTCOG (BAC=66.3%, sensitivity=71.9%, specificity=60.7%) and ROP-POS (BAC=71.5%, sensitivity=71.9%, specificity=71.2%), while slow-4 models significantly differentiated HC from ROP-MOTCOG (BAC=73.7%, sensitivity=70.7%, specificity=76.8%) and from ROP-AFF (BAC=62.1%, sensitivity=72.8%, specificity=51.5%). Functional connectivity significantly differentiated HC from all groups (BAC range: [69.2% - 72.7%], see Table 2). The ROP groups showed a similar deviation profile, characterized by reduced within-default mode network (DMN) coherence with altered coupling to salience/control networks (SN/CN) and increased somatosensory within-network connectivity, but the degree and locus differed: ROP-MOTCOG showed the largest departures (cingulo-opercular/control hubs), ROP-POS more SN abnormalities, ROP-SOCWD a medial-PFC/DMN enhancement, and ROP-AFF a posterior-DMN and cerebellum emphasis (Figure 2B/C/D).

Furthermore, rs-fMRI metrics significantly classified the ROP subgroups against each other (Table 2). Slow-4 fALFF models separated ROP-MOTCOG from ROP-SOCWD (BAC=56.2%, sensitivity=62.5%, specificity=50.0%) and ROP-MOTCOG from ROP-AFF (BAC=68.6%, sensitivity=75.0%, specificity=62.1%), while functional connectivity separated ROP-MOTCOG from ROP-AFF (BAC=68.2%, sensitivity=69.6%, specificity=66.7%). Between-subgroup predictive patterns reflected graded differences in salience–DMN between- and within-network integration in ROP-MOTCOG vs ROP-SOCWD/AFF (Figure 2C/D).

#### Gray matter volume models

All the ROP subgroups showed relatively poor discriminability against the rest of the participants based on GMV data (Figure 1E, Table S11).

Regarding the one-vs-one group models, GMV data separated HC from the ROP-MOTCOG subgroup (BAC=64.3%, sensitivity=57.1%, specificity=71.4%) and the ROP-POS (BAC=61.5%, sensitivity=60.4%, specificity=62.7%) above chance level, while the other two subgroups could not be differentiated from HC (Table 2). For both the ROP-MOTCOG and ROP-POS, model predictive patterns comprised GMV reductions predominantly in fronto-temporal regions (Figure 2E). The ROP subgroups could not be separated from each other using GMV data (Table 2).

#### Genetic models

PRS data best differentiated the ROP-POS subgroup from the rest of the patients and HC, while the other ROP subgroups were poorly separable (Figure 1F, Table S11).

Regarding the one-vs-one models, PRS data separated between HC and ROP-MOTCOG (BAC=62.8%, sensitivity=63.0%, specificity=62.5%) and HC and ROP-AFF (BAC=62.7%, sensitivity=61.8%, specificity=63.6%). The ROP-MOTCOG subgroup was characterized by higher genetic load for schizophrenia, autism and neuroticism relative to HC, while the ROP-AFF was characterized by higher autism and schizophrenia, and lower intelligence genetic load relative to HC (Figure 2F). No genetic separation was found between the ROP subgroups.

#### Multimodal models

Regarding the one-vs-rest models, the ROP-MOTCOG subgroup was the most differentiable based on the multimodal classifier, while the ROP-AFF groups showed the lowest discriminability (Figure 1G, Table S11).

Within the one-vs-one classifications, the multimodal models significantly differentiated HC from all ROP subgroups, as follows: ROP-MOTCOG (BAC=82.1%, sensitivity=74.9%, specificity=89.3%), ROP-POS (BAC=75.9%, sensitivity=72.2%, specificity=79.7%), ROP-SOCWD (BAC=78.0%, sensitivity=74.0%, specificity=82.0%), and ROP-AFF (BAC=73.4%, sensitivity=72.5%, specificity=74.2%). Across subgroups, neurocognition models contributed the most to the multimodal predictive patterns versus HC (Figure 2G). The multimodal classifiers did not survive the FDR-correction in separating between the ROP subgroups, but the models classifying ROP-MOTCOG from all other three subgroups were nominally significant (Table 2).

Furthermore, post-hoc analyses showed moderate correlations between the decision scores of the one-vs-one classifiers separating HC from the ROP subgroups trained on different data modalities, but generally no correlations for the between ROP subgroups classifiers (Table S12).

### Longitudinal functioning trajectories of ROP subgroups

Across both GAF domains, linear mixed-effects models indicated robust main effects of subgroup and time, with no evidence for subgroup×time interactions. Tukey-adjusted contrasts by timepoint showed clear level differences at baseline (T0), driven by lower functioning in ROP-MOTCOG, and parallel improvement from T0 to T2 across all subgroups. Baseline-adjusted endpoint ANCOVAs at T1 and T2 (covarying for T0) were uniformly non-significant, indicating that the predicted labels primarily index initial functional burden rather than differential change over time. For completeness, analyses repeated with the observed PANSS-derived subgroups did not yield significant main or interaction effects (Supplementary Tables S13-S16; Supplementary Figures S5–S8).

## Discussion

Psychoses are clinically diverse, and this heterogeneity has long limited efforts to anchor diagnosis and prognosis in the biology of this disease spectrum [46], [47]. Relating multimodal biology to symptom-based subgrouping in a large multi-site cohort, we show that patients in an early stage of these conditions exhibit distinct clinical “flavours” that share a biological core but diverge through subtle, modality-specific signatures, yielding divergent symptom constellations.

Our factor analysis of PANSS symptoms yielded four subgroups—motor/cognition, positive, social withdrawal, and affective—consistent with prior factorization studies reporting between three and seven domains [7], [9]–[11], [13], [48], [49]. While differences across studies likely reflect methodology and sample variation, our results highlight that the optimal granularity of clinical subtypes for clinical translation should be informed by their biological mapping and prognostic/theragnostic utility.

Across groups, slowed processing speed and functional dysconnectivity consistently emerged as central features, with increased connectivity within sensorimotor networks and reduced within- and between-network connectivity across default-mode, salience, and control systems. This “triple-network” disruption is in keeping with prior literature [50], [51] and may represent a core, symptom-independent network-level disease signature.

Nevertheless, meaningful subgroup distinctions also emerged. The motor/cognition subgroup showed the most pronounced cross-modality separability, being characterized by widespread fronto-temporal GMV loss, marked cognitive and brain activity deficits, poorest functioning throughout the follow-up period, and the highest longitudinal stability, features reminiscent of “deficit schizophrenia” [52]–[54]. The social withdrawal subgroup presented preserved structure but prominent connectivity alterations and lowest social functioning across timepoints, while the affective subgroup exhibited relatively HC-like functional connectivity profiles. The positive subgroup also showed fronto-temporal GMV reductions and network abnormalities, though its separability was lower. These profiles suggest that while functional network changes are broadly shared, structural brain alterations may selectively affect specific subtypes in early stages and potentially foreshadow the fronto-temporal atrophy seen in chronic schizophrenia [31].

Genetical liability for schizophrenia and autism spectrum disorders was enriched across subgroups, consistent with pleiotropic polygenic risk predisposing broadly to psychosis-spectrum phenotypes. Distinct symptom constellations may thus arise from interactions between shared genetic liability, environmental exposures, and developmental timing, shaping downstream brain networks and differentially weighting cognitive, affective, and interpersonal domains [55], [56].

Conceptually, our findings argue against a one-to-one mapping of symptoms to biology and suggest a layered model in which a common neurocognitive–network substrate forms the foundation of early psychosis, while subgroup-specific alterations may arise from partially distinct mechanistic pathways. Thus, some of the subgroups (e.g. Motor/Cognition) may reflect neurodevelopmental liabilities leading to early structural and cognitive impairment, whereas others may represent more dynamic disturbances of brain network function potentially arising through interactions with environmental load and leading to partially overlapping phenotypes through cross-modal interactions.

Clinically, this layered perspective carries implications for patient stratification, such that shared features may serve as general biomarkers of psychosis risk or progression, while subgroup-specific signatures could inform tailored interventions. For instance, the multimodal alterations, clinical stability and functioning impairments of ROP-MOTCOG may justify intensive early interventions, including cognitive remediation and pharmacological support, whereas connectivity-dominant subgroups with preserved structure may be more responsive to psychosocial or behavioral therapies.

### Limitations

Several limitations should be acknowledged. First, subgrouping by maximum factor loading imposes hard boundaries on continuous symptom dimensions. Future work may benefit from modelling individual clinical manifestations as complex interactions and aggregation of these distinct dimensions [57], [58]. Second, we have not explored subgroup-dependent longitudinal brain changes in the current analyses, which could provide further insight into underlying pathophysiological mechanisms. Third, the clinical utility of these bio-behavioral profiles requires validation in prospective studies assessing prediction of treatment response, relapse, and functional outcomes. Lastly, although useful in delineating clinical profiles, PANSS factors may still conflate heterogeneous underlying mechanisms. Thus, defining clinical groups using domain-level, biologically-referenced phenotypes akin to RDoC [4] may offer more direct proxies for biological substrates and further inform psychosis stratification.

## Conclusion

In summary, our findings reveal a shared substrate of neurocognitive and brain network abnormalities overlaid by subgroup-specific, modality-linked alterations in early psychosis. These results provide a nuanced framework for biologically-informed stratification of early psychosis heterogeneity and highlight subgroups of patients with particularly pronounced cross-domain biological burden.

## Methods

### Study Participants

The study adhered to the TRIPOD+ AI reporting guidelines [59] (Transparent Reporting of a Multivariable Prediction Model for Individual Prognosis or Diagnosis; see Supplementary Materials).

Follow-up procedures and the modalities acquired at each assessment point in the longitudinal multi-site PRONIA (Prognostic Tools for Early Psychosis Management; https://www.pronia.eu/) have been detailed in our prior publications [60]. Between /15/2014 and 5/1/2017, 5,547 individuals, covering a catchment population exceeding five million, were screened across seven European sites (München, Udine, Turku, Basel, Birmingham) and, later, three additional sites (Münster, Bari, Düsseldorf). From the total PRONIA sample (see Table S1 for study inclusion and exclusion criteria), 362 ROP patients with available PANSS data were used in the current analyses to derive the factor model (see Figure S1 for specific inclusion criteria). Furthermore, a total of 231 ROP patients and 338 HC individuals had complete data for all investigated data modalities and were used for training the multimodal models (Figure S1). For assessing the longitudinal stability of the factor analysis solution, we used data collected at three time points: baseline (T0), median 9-month (T1), and median 18-month follow-up (T2).

Adult participants gave written informed consent, and patients younger than 18 and their guardians gave written informed assent and consent, respectively. The study was registered at the German Clinical Trials Register (DRKS00005042) and approved by the research ethics committees of all sites.

### Exploratory factor analysis and factor model selection

We analyzed patterns of co-occurring symptoms at baseline (T0) by employing a maximum-likelihood-based exploratory factor analysis (EFA) of the PANSS [8] items using R [61], R Studio [62], and the *psych* (2.1.6) package in R [63]. EFA is a dimensionality reduction technique that aims to find latent dimensions underlying the observed data [64]. As factor loadings depend on the specified number of factors, we compared solutions with one to five factors. To account for correlated symptom dimensions, we used the oblique promax rotation method [65]. We selected the best-fitting model based on the lowest average Bayesian Information Criterion (BIC) after jackknife resampling (n=361) [66]. The BIC is a measure of model fit that introduces a penalty term relative to the number of parameters in the model [67], addressing the issue that more complex models are more prone to overfitting [68]. Jackknife resampling was employed to evaluate the stability of the factor solutions and control for sampling biases on model fit estimates [69]. We tested significant differences between models using paired t-tests and the Mann-Whitney U test between the BIC distributions derived during jackknife resampling. To investigate the stability of the factors over time, we computed the Pearson correlation coefficients (*r*) between item factor loadings across time points and assessed the percentage of items loading highest on the same factor at the different time points using the dice coefficient. Follow-up factor scores were computed by applying the baseline factor model to the follow-up timepoint data.

ROP patients were assigned to subgroups via majority vote during jackknife resampling based on their maximum score on the symptom dimensions of the best-fitting model. Assignment certainty was quantified by how often a subject was assigned to a group relative to another group during jackknife resampling. We assessed the longitudinal stability of the identified symptom dimensions (factor scores) through the relative number of patients assigned to the same symptom group at T1 and T2 respectively, by applying the optimal factor model found at T0, and the Pearson correlations of factor dimensions across time points. We compared the ROP subgroups against each other in terms of the same variables, and the distribution of ICD-10 diagnoses within each group, using ANOVA or Kruskal-Wallis and *X*^2^ tests. Post-hoc comparisons for ROP-subgroups were conducted using Tukey HSD or Dunn tests.

### Neurocognitive data

Neurocognitive performance was assessed by mapping six neurocognitive test measures available in the PRONIA study onto the six domains from the MATRICS consensus cognitive battery: social cognition, working memory, speed of processing, verbal learning, reasoning, and attention [70], [71]. We did not compute the visual learning domain score also included in MATRICS since none of the neuropsychological tests in PRONIA was comparable to the tests used for the computation of the domain score. Additionally, we computed one composite score for global cognition. Table S2 gives an overview of the neuropsychological tests included in the computation of the cognitive domain scores.

### Acquisition and preprocessing of MRI data

Structural neuroimaging data was available for 308 ROP patients and 440 HC individuals and rs-fMRI data was available for 295 ROP patients and 428 HC individuals, following quality-based exclusion criteria (Figure S1). The PRONIA study aimed to capture the natural heterogeneity of MRI sequences encountered in clinical settings and, therefore, required minimal harmonization of magnetic resonance imaging (MRI) scanners across sites. Site-specific acquisition parameters for the sMRI and rs-fMRI data are presented in Table S3 and S4, respectively.

Acquired T1-weighted images were visually inspected, anonymized, and defaced using an in-house script based on the Freesurfer toolbox. Subsequently, structural MRI scans were pre-processed with the open-source CAT12 toolbox (version r1207; http://dbm.neuro.uni-jena.de/cat12/), an extension of SPM12 using a standardized pipeline. Images were segmented into white matter, gray matter, and cerebrospinal fluid and registered to the MNI-152 space using the DARTEL algorithm [72]. Gray matter tissue maps were modulated using the Jacobian determinants from the registration step, producing GMV maps in standard space. These GMV maps were smoothed using a 4mm^3^ full-width-at-half-maximum Gaussian kernel. Image quality checks were done by applying CAT12’s quality assurance framework [73], which produces a score from excellent (A) to failed (F) for each image. Images of nine participants with a score of C (satisfactory) or lower were excluded from the analysis. Finally, the data was proportionally scaled using the total intracranial volume.

For the rs-fMRI domain, data were preprocessed using Statistical Parametric Mapping (SPM12, version 6685; http://www.fil.ion.ucl.ac.uk/spm) in combination with the Resting-State fMRI Data Analysis Toolkit (REST, version 1.848; http://www.restfmri.net/), following a modified version of a pipeline previously developed within the PRONIA consortium [70]. The preprocessing procedure included removal of the initial 8 volumes, slice timing correction, realignment, co-registration with structural T1-weighted images, resampling, normalization to MNI space, nuisance regression (including white matter, CSF, and the Friston 24 head motion parameters), spatial smoothing using a 6 mm FWHM Gaussian kernel, motion artifact correction via despiking, and linear detrending. Following this, two rs-fMRI-based metrics were extracted: (1) voxel-wise fractional amplitude of low-frequency fluctuations (fALFF), obtained by applying a fast Fourier transform to the preprocessed time series and computing the ratio between the power in a specific low-frequency band (slow-5 (0.01–0.027 Hz) and slow-4 (0.027–0.073 Hz), based on previous literature [74] and the total power across the full frequency spectrum. Additionally, fALFF values were Z-standardized across voxels for each individual; (2) functional connectivity, computed as partial correlation coefficients between the band-filtered time series (0.01–0.1 Hz) extracted from 176 regions of interest (ROI) from the Dosenbach [75] and SUIT [76] functional atlases.

### Genetic data

Genotyping was performed using Illumina’s Infinium Global Screening Array-24 BeadChip version 2 with additional psychiatric content (GSA), comprising over 650,000 markers, including 50,000 variants associated with psychiatric disorders such as schizophrenia, bipolar disorder, and autism spectrum disorder. Following stringent quality control using PLINK (sample and variant call rate > 0.98; minor allele frequency > 0.01; Hardy-Weinberg equilibrium P<1×10^−6^; sex verification; heterozygosity filtering), 505,687 variants remained. The cleaned genotype data were phased using Eagle v2.4.1 and imputed with Minimac4 v1.0.2, employing the 1000 Genomes Phase 3 dataset as a reference panel. Variants with imputation quality (R^2^) below 0.8 (n=10,962,225 variants) were excluded. Polygenic risk scores (PRS) for neuropsychiatric, cognitive, and behavioral traits (listed in Table S5 were computed using the PRS-CS method with the auto setting and default hyperparameters. PRS-CS employs a Bayesian continuous shrinkage model, integrating GWAS summary statistics with linkage disequilibrium (LD) data from the UK Biobank European LD reference panel (ldblk_ukbb_eur), enabling genome-wide PRS estimation without relying on clumping or p-value thresholds. All resulting PRS values were standardized across participants to facilitate comparability. To account for population stratification, the first ten principal components (PCs) were derived from LD-pruned genotype data (window size=50 variants, step size=5 variants, R^2^<0.5).

### Multivariate biological characterization of symptom-based subtypes

We trained multi-class machine learning models using the open-source NeuroMiner (version 1.3; https://github.com/neurominer-git) toolbox in order to differentiate the HC individuals and the four ROP subgroups against each other within a repeated nested cross-validated (CV) framework with 10 folds and 5 permutations on both the inner and outer CV layers with a one-vs-one multi-class decomposition approach. Models were trained on individual data modalities (uni-modal), as well as by stacking uni-modal models into multimodal models.

Modality-specific preprocessing steps were embedded within the cross-validation structure and included: (1) neurocognition: covariate regression of age and sex, standardization and winsorization of outliers over ± 4 standard deviations; (2) rs-fMRI metrics: site correction using mean offset adjustment and covariate regression of age, sex and mean framewise displacement based on the HC individuals, thresholding the maps using G-theory-based inter-site reliability maps, principal component analysis for dimensionality reduction (retaining [40%, 60%, 80%] of the total energy decomposition, optimized within the inner CV layer) and median-based standardization and winsorization of outliers over ± 4 standard deviations; (3) GMV: the same steps as for the rs-fMRI data, and additional smoothing with a FWHM Gaussian kernel optimized between 4-6-8 mm; (4) genetic data (PRS): covariate regression of age and sex and the first 10 ancestry components, standardization and winsorization of outliers over ± 4 standard deviations.

Subsequently, uni-modal multiclass L2-regularized support vector machine (SVM) classifiers (LIBLINEAR, https://www.csie.ntu.edu.tw/~cjlin/liblinear/) were trained by optimizing for the multi-class problem by means of Error-Correcting Output Codes using the Hamming distance between the predicted and reference codes [77]. The slack parameter was optimized in geometric progression between 2^−6^ and 2^4^ and we used hyperplane weighting for adjusting class imbalance. Subsequently, using the same nested CV approach, we trained a multimodal stacked model combining the models trained on individual data modalities in order to assess their complementary predictive value. We assessed model significance against chance using a permutation-based procedure (1000 iterations), comparing the observed balanced accuracy (BAC) with an empirical null distribution generated by shuffling group labels and re-estimating out-of-training BAC. Furthermore, we visualized predictive patterns using the cross-validation ratio (CVR) as a measure of feature stability. As reported in detail in previous publications from our group [60], the CVR is computed by calculating the mean and standard error of all SVM weight vectors concatenated across the entire nested cross-validation structure, similar to the bootstrap ratio approach [78].

Given the one-vs-one multi-class approach, we report both averaged results of one-vs-rest comparisons (each subgroup vs. all other patients and HC), as well as all one-vs-one classifiers. Additionally, we ran post-hoc correlation analyses investigating the associations between the decision scores of classifiers trained on different data modalities across the binary one-vs-one models.

### Longitudinal functioning trajectories

We investigated functioning using the Global Assessment of Functioning symptom (GAF-S) and disability (GAF-DI) scales assessed at baseline (T0) and follow-up (T1, median 9 months; T2, median 18 months). The primary exposure was subgroup membership derived from the out-of-fold (OOF) predictions of the multimodal multi-class classifier described above (ROP-MOTCOG, ROP-POS, ROP-SOCWD, ROP-AFF). Using OOF labels ensured that each participant’s subgroup assignment was generated without information leakage from their own data. As a sensitivity analysis, the same models were repeated with the observed PANSS-derived subgroups; these results are summarized in the Supplement. For the longitudinal analysis, we fitted linear mixed-effects models with participant-specific random intercepts to account for repeated measures across T0–T2. Fixed effects included Subgroup, Time, and their interaction, with age, and sex as covariates. We report Type-III Wald χ^2^ tests for main and interaction effects, estimated marginal means (EMMs) by subgroup and timepoint with Tukey-adjusted pairwise comparisons, and intraclass correlation coefficients (ICC) derived from model variance components to quantify within- versus between-person variance. Model assumptions were assessed using our standard diagnostics pipeline (distributional checks of residuals and over/under-dispersion, collinearity screening via variance inflation factors). Analyses used available cases without imputation. To evaluate level differences at follow-up beyond baseline, we additionally conducted baseline-adjusted ANCOVAs with T2 GAF-S or GAF-DI as the dependent variable, entering the corresponding T0 score as a covariate alongside age, and sex. We present Type-III tests for the subgroup effect at T2, adjusted EMMs, and Tukey-corrected pairwise contrasts. All analyses were performed in R (version 4.5.1, [79]), using lme4 [80] for mixed models and emmeans for marginal means and contrasts [81]. Two-sided tests were used throughout with α=.05, and multiplicity was controlled at the contrast level via Tukey adjustment.

## Supplementary Material

Supplementary Files

This is a list of supplementary files associated with this preprint. Click to download.

- SupplementMultimodBiologicalProfilesROPsubgroups.doc
- Table23.docx

## Figures and Tables

**Table 1.** **Statistical comparison of sociodemographic, cognitive, and clinical characteristics** between the HC individuals and ROP patients with all modalities, as well as between the four ROP subgroups at baseline.

Variable	Whole groups				4-Factor Solution					
	HC	ROP	Test statistic	FDR *P*	ROP-MOTCOG	ROP-POS	ROP-SOCWD	ROP-AFF	Test statistic	FDR *P*
**Sociodemographic**										
N	338	231			56	59	50	66		
Age(SD)	25.35 (5.86)	25.90 (5.66)	W=36815.5	.250	25.00 (5.11)	25.74 (6.37)	26.69 (5.30)	26.29 (5.77)	H=3.5	.786
Sex, male/female (%)	139/199 (41/59)	133/98 (58/42)	χ^2^=14.2	<.001	35/19 (65/35)	33/26 (56/44)	30/19 (61/39)	32/34 (48/52)	χ^2^=3.7	.786
Education, yrs (SD)	15.80 (3.35)	14.47 (7.99)	W=50933	<.001	13.25 (3.32)	13.84 (2.91)	14.07 (2.89)	16.26 (14.01)	H=6.9	.611
**Clinical**										
**Family psychosis risk**										
1^st^ degree (%)	0 (0)	37 (16)	Fisher’s exact	<.001	7 (13)	10 (17)	10 (20)	9 (14)	Fisher’s exact	.799
2^nd^ degree (%)	11 (3.3)	27 (12)	Fisher’s exact	<.001	4 (7.4)	10 (17)	5 (10)	8 (12)	Fisher’s exact	.786
Head Trauma (%)	28 (8.3)	52 (23)	Fisher’s exact	<.001	13 (24)	11 (19)	14 (29)	13 (20)	Fisher’s exact	.786
Birth Complications (%)	40 (12)	40 (17)	Fisher’s exact	.065	5 (9.3)	13 (22)	11 (22)	10 (15)	Fisher’s exact	.786
Onset Age (SD)		25.62 (5.66)			24.50 (5.14)	25.57 (6.18)	26.21 (5.37)	26.25 (5.89)	H=3.3	.786
Illness Duration, years (SD)		0.55 (0.54)			0.61 (0.57)	0.40 (0.42)	0.69 (0.65)	0.51 (0.51)	H=8.3	.611
PANSS Total		68.8 (19.4)			69.06 (22.68)	71.19 (20.61)	68.41 (15.07)	65.73 (17.03)	H=1.9	.786
**Treatment**										
Ever Psychotherapy (%)	54 (16)	139 (60)	Fisher’s exact	<.001	28 (52%)	35 (59%)	31 (63%)	44 (67%)	Fisher’s exact	.786
Ever Hospitalization (%)	9 (2.7)	165 (71)	Fisher’s exact	<.001	41 (76%)	40 (68%)	31 (63%)	50 (76%)	Fisher’s exact	.786
Ever Pharmacotherapy (%)	6 (1.8)	197 (85)	Fisher’s exact	<.001	46 (85%)	50 (85%)	42 (86%)	56 (85%)	Fisher’s exact	.989
**Medication intake**										
Antipsychotics (CPZE-Equiv.)		700.89 (3965.69)			258.09 (145.34)	228.86 (176.44)	227.33 (202.66)	269.07 (385.67)	H=2.1	.786
OLA-Equiv.		24.09 (132.27)			8.86 (5.01)	7.89 (6.36)	7.91 (6.99)	72.38 (334.12)	H=2.3	.786
Antidepressants (SSRI-Equiv.)		19.62 (167.69)			25.60 (5.86)	22.26 (14.24)	29.78 (17.62)	31.56 (17.82)	H=0.9	.893

*Note*. CPZE, Chlorpromazine; GF, Global Functioning; GAF; Global Assessment of Functioning (D/I, Disability and Impairment; S, Symptoms); SD, Standard Deviation; SSRI, Selective serotonin reuptake inhibitor; Significance threshold defined at *P*<.05.

Cumulative Dosage [mg].

Table 2 and 3 are available in the Supplementary Files section.

## Data Availability

Due to restrictions in participants’ informed-consent agreements, the PRONIA dataset cannot be made public. The trained machine learning models can be obtained from the corresponding author upon request. The NeuroMiner toolbox used for training all the machine learning classifiers is available online at https://github.com/neurominer-git (version 1.3).
